# Prospective Cohort Study to Elucidate the Correlation between Occupational Stress and Hypertension Risk in Oil Workers from Kelamayi City in the Xinjiang Uygur Autonomous Region of China

**DOI:** 10.3390/ijerph14010001

**Published:** 2016-12-22

**Authors:** Rong Li, Xiaoyan Gao, Bo Liu, Hua Ge, Li Ning, Junling Zhao, Jiwen Liu

**Affiliations:** 1 Department of Public Health, Xinjiang Medical University, Urumqi 830011, China; lurong_128@126.com (R.L.); 15199142607@163.com (X.G.); liubo1021@sina.cn (B.L.); gehua2710@sina.com (H.G); nl96979@163.com (L.N.); zhjl8899@sina.com (J.Z.); 2 The First Affiliated Hospital of Xinjiang Medical University, Urumqi 830011, China

**Keywords:** oil workers, occupational stress, hypertension, cohort study

## Abstract

The purpose of this study was to assess the major risk factors for hypertension in oil workers, and investigate the effect of occupational stress on the incidence of hypertension after controlling for other risk factors. A prospective cohort approach was used following enrollment of 1354 oil workers. The occupational stress experienced by oil workers was higher than for the general population in China. By the end of the cohort study, 231 new cases of hypertension among the oil workers had been diagnosed. The cumulative incidence of hypertension was 17.06%. There were 44, 112, and 75 workers who developed hypertension in the low, intermediate, and high occupational stress groups, which represented a 12.0%, 15.6%, and 20.3% cumulative incidence, respectively (chi-square value = 9.812, *p* < 0.01). Multivariate Cox proportional hazard model analysis showed that type of work, cigarette smoking, excess body weight, and obesity were risk factors for hypertension (*p* < 0.05). After risk factors such as type of work, cigarette smoking, alcohol consumption, and body mass index (BMI) were controlled, the hypertension risk (hazard ratio, HR) in the high occupational stress group was 1.549 (1.072–2.236) compared to the low exposure group, and 2.337 (1.191–4.585) in female subjects. Our study indicated that an increase in occupational stress was associated with an increased risk of hypertension after other factors were adjusted.

## 1. Introduction

Hypertension is a major global health issue due to its magnitude and associated risks, difficulty of control, high medical and social costs, and the fact that it causes severe cardiovascular and renal complications [1]. In 2010, hypertension caused 9.4 million deaths and led to the loss of 7% disability-adjusted life years [2]. It has been proposed that non-communicable cardiovascular diseases (CVDs) will be the global leading cause of death and disability by 2020 [3], and the number of hypertensive adults will reach 1.5 billion (approximately 30% of the world population) by 2025 [4]. In addition to known risk factors for hypertension, such as prior family history, obesity, tobacco use, alcohol consumption, and a high intake of sodium [5]; multiple factors, including age, gender, blood glucose, triglycerides, total cholesterol, high-density and low-density lipoprotein levels, are also closely correlated with the incidence of hypertension [6,7]. While genetic and behavioral factors leading to hypertension have been intensely studied, the etiology of hypertension is still largely unknown.

In recent years, agricultural and industrial advances have impacted lifestyle and social interaction, leading to greater psychological pressure. Epidemiologic studies have shown the workplace to be a high-pressure environment. The imbalance between work demand and work control in modern society is considered to be an important risk factor for hypertension in men [8] and women [9]. A recent study has demonstrated that long-term and excessive occupational stress promotes a number of unhealthy behaviors, such as cigarette smoking, alcohol consumption, high-fat diets, drug abuse, and a sedentary lifestyle [10], which are all risk factors for hypertension.

Occupational stress combined with other risk factors is associated with a higher incidence of hypertension compared to occupational stress alone [11]. Furthermore, there is an interaction between occupational stress and other risk factors, such as gender, age, alcohol consumption, and obesity, leading to hypertension [11]. A cohort study conducted by Wiernik et al. [12,13], which included 122,816 adults, found that for women with a relatively low occupational status, occupational stress is a potential risk factor for hypertension. Additionally, the effects of stress are being studied in additional areas, such as marriage and low socioeconomic status. Associations between these other risk types and blood pressure (BP) outcomes have been reported, but the results vary, and ongoing exposure to stress may be more plausibly linked to sustained BP elevation and incidence of hypertension [14]. However, occupational stress has also been identified as an independent risk factor for hypertension [15,16].

Previous studies often applied a cross-sectional approach and short-term follow-up to explore the relationship between occupational stress and blood pressure. In China, relevant studies have only recently been completed and cross-sectional studies have been limited. In addition, the cultural background, labor organization, and sources of stress in China differ from other countries. Therefore, the role of occupational stress in hypertension amongst the Chinese working population warrants more specific studies. The objective of this study was to investigate the correlation between occupational stress and hypertension in oil workers in Kelamayi City through a prospective cohort study.

## 2. Materials and Methods

### 2.1. Population and Study Design

This prospective cohort study enrolled oil workers from seven oil companies in Kelamayi City. The data were collected at two time points (baseline (2013–2014) with a follow-up period of two years (2014–2016). Baseline data included blood pressure (taken at the Central Hospital of Kelamayi City, China), and a questionnaire assessing occupational stress state and hypertension risk factors was completed by the subjects. The inclusion criteria were as follows: (1) oil workers from one of the seven companies who had physical examinations in the hospital between 2013 and 2014 and completed the occupational stress questionnaire; and (2) voluntary consent to participate in the study after being informed of the objective and significance of the study (informed consent). The exclusion criteria were as follows: (1) previous diagnosis of hypertension; (2) family history of hypertension; (3) rejection of follow-up; and (4) incomplete relevant information and data. The diagnostic criteria of new hypertension cases were based on the *Chinese Hypertension Prevention and Treatment Guides: Version 2004* [17]. New hypertension cases were considered to be when subjects with no history of hypertension and physical examination in 2013–2014 showing a systolic blood pressure (SBP) < 140 mmHg and a diastolic blood pressure (DBP) < 90 mmHg, had a physical examination in 2015 showing a SBP ≥ 140 mmHg or DBP ≥ 90 mmHg, or alternatively were diagnosed with hypertension in a qualified hospital and administered anti-hypertensive drugs. Diagnosis of hypertension was considered to be the ending event, and the investigators allowed the subjects to complete the questionnaire (the same as at baseline) according to their situation in the past year. The subjects who did not have an ending event during their follow-up were asked to complete the questionnaire at the end of the cohort study. The baseline number of subjects was 1451, and 97 subjects (7%) were censored at the end of the follow-up period. The reasons for censoring were primarily due to occupational changes or retirement. A total of 1354 subjects, including 715 males (52.8%) and 639 females (47.2%), completed the whole study. Two hundred and thirty-one subjects (17.1%) were newly-diagnosed with hypertension.

### 2.2. Occupational Stress Evaluation

The OSI-R Scale (Occupational Stress Inventory, revised edition, OSI-R) was used to evaluate the occupational stress of study participants. The scale was originally developed by Osipow in 1981 [18] and revised seven times thereafter. Li et al. [19] introduced the scale to China in 1998, and after revision and verification, the scale has been shown to have high reliability and validity. The OSI-R scale has been widely used in studies of occupational stress in China and includes the following three sections: Occupational Role Questionnaire (ORQ); Personal Strain Questionnaire (PSQ); and Personal Resources Questionnaire (PRQ). The scale sections are used to measure occupational stress, psychological strain, and coping resources of the subjects, respectively, in an effort to comprehensively reflect the occupational stress of the subjects.

OSI-R is a multiple-dimension scale. Each item has five Likert-type scales (never, seldom, sometimes, frequently, and always). The raw scores range from 1–5. The higher the score of the ORQ and PSQ, the higher the occupational stress and psychological strain; the higher the score of the PRQ, the higher the capacity of the subject to cope with stress. For ORQ and PSQ scales, higher scores are associated with a high level of occupational stress. Grouping methods used to divide participants into low, intermediate, and high levels were set according to the corresponding references [20]. Specifically, the scores were as follows: occupational stress (low, <258; intermediate, 258–307; and high, >307); occupational role (low, <161; intermediate, 161–194; and high, >194); personal strain (low, <89; intermediate, 89–116; and high, >116); and personal resources (low, <105; intermediate, 105–134; and high, >134). In the present study, Cronbach’s alpha coefficients of all of the scales and dimensions were >0.70, which indicated that the OSI-R had good reliability.

### 2.3. Blood Pressure Measurement

Blood pressure was measured by a physician using an electronic blood pressure monitor. Before measurement, the subjects were asked to remain seated for at least 15 min. The right upper arm SBP and DBP were measured twice for each subject. There was a 2-min interval between the two measurements, and the final blood pressure was the average value of the two measurements. If the blood pressure was ≥140/90 mmHg, the subject was asked to rest for 15 min, BP was retaken twice, and the average of the two measurements was the final result. The above measurement method was performed according to the *China Chronic Disease Surveillance Project* [21], published by the National Work Group of the project.

### 2.4. Covariates

Cigarette smoking, alcohol consumption, body mass index (BMI), salt intake, and a sedentary lifestyle are considered to be risk factors for hypertension. Smoking status was categorized as follows: non-smoker; previous smoker (already quit, and quit ≥1 year); and current smoker (at least 1 cigarette per day for >1 year). Alcohol consumption status was categorized as follows: none; occasional; and regular consumption (at least twice per week for >1 year). Physical exercise status was categorized as follows: none; occasional; and regular (≥3 times per week for ≥30 min each session). BMI was categorized as follows, according to the criteria of the *Chinese Adult Overweight and Obesity Prevention and Control Guideline* [22]: BMI < 24 kg/m^2^ was considered as “normal weight”, BMI ≥ 24 kg/m^2^ as “overweight” and BMI ≥ 28 kg/m^2^ as “obesity”. In addition, some key demographic characteristics, such as age, gender, ethnicity, educational level, number of years worked, marital status, income, and work shift, were also included. In the current study, these factors were considered to be confounding factors and controlled in the analysis.

### 2.5. Statistical Analyses

Numeric data with a normal distribution were presented as the mean ± standard deviation. Comparison of the means of two groups was performed using an independent sample *t*-test. ANOVA was used for comparison of the means of multiple groups. If the overall difference was significant, then a least significant difference *t*-test was used for pairwise comparisons. Count data are presented as a percentage (n %). A Chi-square test was used for inter-group comparisons and the effect of occupational stress on the risk of hypertension was analyzed using a multivariate Cox proportional hazards regression model. The significance level (α) was set at 0.05. The data were analyzed using SPSS 17.0 (IBM, Armonk, NY, USA).

## 3. Results

### 3.1. General Demographic Characteristics of the Subjects

The general demographic characteristics of the subjects are shown in Table 1.

### 3.2. Score of Occupational Stress

#### 3.2.1. Score of Occupational Stress in Oil Workers and Chinese Norm

The results demonstrated that the occupational role, personal strain and personal resource scores of oil workers were significantly higher than the Chinese norm (*p* < 0.05; Table 2).

#### 3.2.2. Score of Occupational Stress in Different Type of Work Groups

The results showed that the scores of occupational role, personal strain and personal resources were not significantly different between the types of work groups (*p* > 0.05; Table 3).

### 3.3. Incidence of Hypertension

#### 3.3.1. Incidence of Hypertension in the Subjects with Different Characteristics

At the end of the cohort study (April 2016), there were 231 (17.06%) new cases of hypertension in 1354 follow-up subjects. The number of isolated systolic hypertension, isolated diastolic hypertension, and systolic-diastolic hypertension cases was 21, 113 and 97, respectively (Table 4). The age, number of years worked, and BMI of the subjects with hypertension were all significantly higher than for the subjects without hypertension (*p* < 0.01). Moreover, there were significant differences in gender, cigarette smoking history, alcohol consumption history, and BMI between the hypertensive and non-hypertensive groups (*p* < 0.05). With respect to type of work, no significant difference was observed between the two groups with respect to work shift or physical exercise (*p* > 0.05; Table 5). The results suggested that gender, number of years worked, cigarette smoking, alcohol consumption, and BMI might be associated with hypertension in oil workers.

#### 3.3.2. Scores of Occupational Stress of Subjects with and without Hypertension

The results showed that the occupational role and personal strain scores of the subjects with hypertension were significantly higher than for subjects without hypertension (*p* < 0.05). With respect to personal resources, there was no significant difference between the two groups (*p* = 0.504; Table 6).

#### 3.3.3. Incidence of Hypertension in Different Occupational Stress Groups

**Figure 1:**
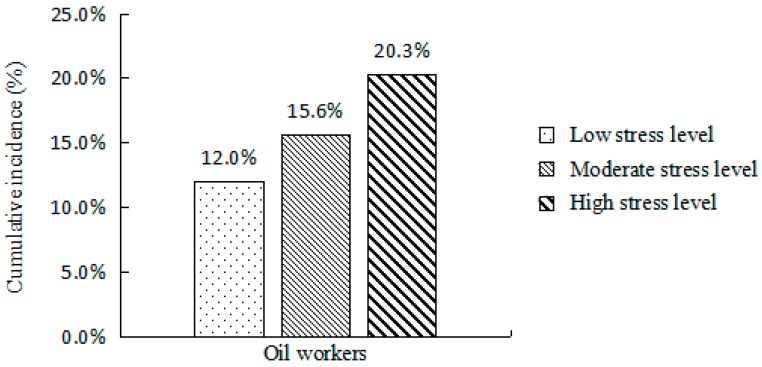
Two-year cumulative incidence (%) in different occupational stress groups.

The results demonstrated that there were 44, 112, and 75 patients with hypertension in the low, intermediate, and high-occupational stress groups, representing a 12.0%, 15.6%, and 20.3% cumulative incidence, respectively. The incidence of hypertension was positively correlated with the level of occupational stress and the straight trend had statistical significance (Chi-square value = 9.812, *p* < 0.01; Figure 1).

### 3.4. Cox Proportional Hazards Model of Hypertension Risk

A multivariate Cox proportional hazards model showed that type of work, cigarette smoking, excess body weight, and obesity were risk factors for hypertension, but occasional drinking was a protective factor for hypertension (*p* < 0.05; Table 7).

### 3.5. Correlation between Occupational Stress and Risk for Hypertension

In the current study we constructed two models to determine the relationship between occupational stress and risk for hypertension. Based on the OSI-R scores, the variable representing occupational stress was entered into the models for analysis. Model 1 is a univariate model in which the different levels of occupational stress are independent variables; the group with the lowest level served as the reference group and the incidence of hypertension is a dependent variable. The subjects with a high occupational stress had a higher risk for hypertension; the HR was 1.675 (1.161–2.418; *p* < 0.05). Model 2 is a multivariate model that was constructed by adjusting the factors that may be related to the risk for hypertension (confounding factors). The HR in the high occupational stress group was 1.549 (1.072–2.236; *p* < 0.05). The risk for hypertension in the subjects with high occupational stress was 1.549-fold greater than subjects with low occupational stress (Table 8). According to gender stratification, the female subjects with high occupational stress had a higher risk for hypertension; the HR was 2.208 (1.133–4.303) in model 1 (*p* < 0.05), the HR in the high occupational stress group was 2.337 (1.191–4.585) in model 2 (*p* < 0.05; Table 9). However, there was no significant difference in risk for hypertension in different occupational stress groups in male subjects (Table 10).

## 4. Discussion

Oil workers in Kelamayi City of Xinjiang, China, have higher occupational stress and a high intensity of work that is associated with abnormal blood lipids, blood glucose, and immune function [23,24]. In the current study, we identified that the occupational stress of oil workers was higher than the Chinese general population [25], with a total of 231 subjects developing hypertension during the study period, thus representing a 17.06% cumulative incidence. Wang et al. reported that the prevalence of hypertension was 29.6% in the Chinese general population in 2009 [26] and a cohort study by Wiernik et al. reported that in France the prevalence of hypertension was 27.0% from 1996 to 2007 [12]. The study participants who were male, older, worked a long number of years, smoked cigarettes, consumed alcohol, and were overweight had a higher incidence of hypertension. Previous studies have shown that the incidence of hypertension is primarily associated with age, gender, BMI, cigarette smoking, and genetic factors [27,28]. In this study there were 44, 112, and 75 new cases of hypertension in the low, intermediate, and high occupational stress groups, which represented a 12.0%, 15.6%, and 20.3% cumulative incidence, respectively. The scores of occupational role and personal strain of the subjects with hypertension were significantly higher than for subjects without hypertension. Moreover, the incidence of hypertension was positively correlated with occupational stress. Our study is in agreement with previous studies [29,30] and further supports the notion that high occupational stress in oil workers could increase the risk of hypertension. A multivariate Cox proportional hazards model showed that type of work, cigarette smoking, excess body weight, and obesity are risk factors that increase the incidence of hypertension. After adjustment for confounding factors, such as type of work, cigarette smoking, alcohol consumption, and BMI, the HR of high occupational stress was 1.549 (1.072–2.236) compared to the low-exposure group, and 2.337 (1.191–4.585) in female subjects. Our results are consistent with Wiernik et al. [12], who included 122,816 adult subjects (average age, 46.8 ± 9.9; and male subjects, 69.2%) and concluded that occupational stress is associated with the incidence of hypertension (OR = 1.06, 95% CI = 1.03–1.09) after other factors, except occupational status, were adjusted.

Why do the oil workers have the high prevalence of both hypertension and occupational stress? With the increase in the demand of oil in China, the expansion of personnel cannot satisfy the demand, which leads to a continuous increase in the workload of oil workers. The working conditions for oil workers can be harsh, working in an oilfield that is far from a city and often in the desert requires they often live in the staff dormitory for work in the field, leading to long-term separation from families and development of negative psychological states including loneliness, anxiety, and depression. Meanwhile, mechanical automation is constantly improving, requiring oil workers to acquire new knowledge and techniques by enrolling in training courses in their spare time, further increasing their physical and psychological stress. An unhealthy lifestyle, such as irregular diet, excessive alcohol consumption, cigarette smoking, and other unhealthy behaviors, is also common in the oil worker population. All of these factors have a negative impact on the physical and mental health of oil workers. In severe circumstances, the workers may develop sleep disorders, physical pain, or other symptoms seriously affecting their work and lives.

We recognize that there are limitations associated with our study. The follow-up time was relatively short and approximately 7% of subjects were censored, which may have influenced the results. Moreover, hypertension is associated with genetic and environmental factors. In this study, we focused our investigation on occupational stress (occupational psychological factors in the work environment), with genetic factors as well as the interaction between genetic factors and the environment not covered. Furthermore, occupational stress is a form of chronic psychological stress, promoting constant stimulation of the hypothalamus-pituitary-adrenal (HPA) axis leading to accelerated secretion of adrenaline, norepinephrine and other substances. These substances can increase blood pressure, heart and respiratory rate, and when secreted and accumulated in an abnormal manner this can lead to metabolic disorders.

Important advantages of this study include the following: (1) The subjects were oil workers in a special work environment, and the sample size was large; (2) Preliminary studies provided reliable supporting data for the present study; (3) In addition to occupational stress, the current study also investigated other lifestyle factors that may affect the incidence of hypertension. By controlling other confounding factors, the study clarified the relationship between occupational stress and hypertension.

The correlation between occupational stress and the pathogenesis of hypertension revealed in this study may have an important impact on the health of the population. At the population level, a decrease of 2 mmHg from the average SBP reduces the number of deaths due to stroke and cardiovascular diseases by approximately 10% and 7%, respectively [31]. Therefore, applying active and effective measures to reduce the levels of these risk factors will have a significantly beneficial effect on public health.

## 5. Conclusions

Lifestyle behaviors (cigarette smoking, alcohol consumption, and BMI) and occupational stress (occupational role and individual strain) are major risk factors for hypertension in oil workers. After controlling for other factors except occupational stress, our results suggest that an increased level of occupational stress could increase the risk of hypertension. Occupational stress may be an independent risk factor for hypertension in oil workers; however, this conclusion needs to be verified by further large-size samples and longer follow-up studies.

## Figures and Tables

**Table 1:** General demographic characteristics of the subjects.

Items	Groups	Case Number	Percentage (%)
Gender	Male	715	52.8
	Female	639	47.2
Age (years old)	<35	389	28.7
	35–45	563	41.6
	45–60	402	29.7
Number of working years	≤20	630	46.6
	>20	724	53.4
Work shift	Fixed day shift	573	42.3
	Work in shifts	781	57.7
Ethnicity	Han	1064	78.5
	Minority	290	21.5
Educational level	High school or less	316	23.3
	Secondary technical school and college	728	53.8
	University and above	310	22.9
Marital status	Unmarried	136	10.0
	Married	1087	80.2
	Divorced or widowed	131	9.8
Income (yuan)	≤3500	508	37.5
	>3500	846	62.5

**Table 2:** Comparison of the score of occupational stress between oil workers and Chinese norm.

Items	Oil Workers	Chinese Norm	t Value	*p* Value
Occupational role	176.87 ± 28.85	162.89 ± 27.04	16.455	<0.001
Personal strain	103.53 ± 20.82	91.01 ± 17.19	22.12	<0.001
Personal resources	119.21 ± 23.57	129.23 ± 17.73	−15.638	<0.001

**Table 3:** Comparison of the score of occupational stress in different types of work groups.

Groups	Case Number	Occupational Role	Personal Strain	Personal Resources
Oil production workers	501	179.32 ± 26.02	103.32 ± 21.55	123.29 ± 22.40
Oil transportation workers	338	177.97 ± 28.74	105.08 ± 20.62	123.94 ± 20.81
Oil refining workers	359	178.62 ± 30.43	103.19 ± 19.04	124.33 ± 21.11
Other workers	156	182.48 ± 27.13	103.81 ± 21.65	120.09 ± 22.66
t value		0.983	0.628	2.466
*p* value		0.400	0.597	0.061

**Table 4:** Comparison of the characteristics of subjects with and without hypertension. BMI: body mass index.

Type of Hypertension	Case Number	Percentage (%)
Isolated systolic hypertension	21	9.09
Isolated diastolic hypertension	113	48.92
Systolic-diastolic hypertension	97	41.99
Total	231	100

**Table 5:** Comparison of the characteristics of subjects with and without hypertension.

Items	Hypertension (*n* = 231)	Non-Hypertension (*n* = 1123)	t/Chi-Square Value	*p* Value
Age (years old)	43.25 ± 6.22	39.06 ± 7.45	8.991	<0.001
Number of years worked	23.77 ± 9.29	17.99 ± 9.12	8.509	<0.001
BMI (kg/m^2^)	26.58 ± 8.11	24.13 ± 11.10	3.188	0.001
Gender			38.756	<0.001
Male	165 (23.08)	550 (76.92)		
Female	66 (10.33)	573 (89.67)		
Type of work			0.831	0.842
Oil production workers	80 (15.97)	421 (84.03)		
Oil transportation workers	60 (17.75)	278 (82.25)		
Oil refinery workers	65 (18.11)	294 (81.89)		
Other workers	26 (16.67)	130 (83.33)		
Work shift			2.703	0.108
Fixed day shift	109 (19.02)	464 (80.98)		
Rotation shift	122 (15.62)	659 (84.38)		
Smoking history			61.454	<0.001
No	84 (10.92)	685 (89.08)		
Previous	44 (18.18)	198 (81.82)		
Often	103 (30.03)	240 (69.97)		
Drinking history			94.742	<0.001
No	76 (12.97)	510 (87.03)		
Occasional	78 (13.31)	508 (86.69)		
Often	77 (42.31)	105 (57.69)		
Physical exercise			0.355	0.837
No	56 (17.72)	260 (82.28)		
Occasional	139 (16.59)	699 (83.41)		
Often	36 (18.00)	164 (82.00)		
BMI			100.498	<0.001
Normal	55 (7.85)	646 (92.15)		
Overweight	108 (23.48)	352 (76.52)		
Obesity	68 (35.23)	125 (64.77)		

**Table 6:** Comparison of the occupational stress scores of subjects with and without hypertension.

Groups	Occupational Role	Personal Strain	Personal Resources
Hypertension (*n* = 231)	188.03 ± 24.15	109.37 ± 21.60	123.60 ± 20.76
Non-hypertension (*n* = 1123)	177.34 ± 28.46	102.63 ± 20.30	122.54 ± 22.22
t value	−0.5330	−4.542	−0.668
*p* value	<0.001	<0.001	0.504

**Table 7:** Analysis of the risk factors for hypertension.

Independent Variables	β	SE	Chi-Square Value	*p* Value	HR (95% CI)
Gender	−0.269	0.165	2.649	0.104	0.764 (0.553–1.056)
Age (years old)	0.029	0.021	1.996	0.158	1.029 (0.989–1.072)
Work years	0.025	0.016	2.557	0.108	1.026 (0.994–1.058)
Work shift	0.123	0.152	0.657	0.418	1.131 (0.840–1.522)
Type of work	-	-	32.890	<0.001	-
Oil production workers	0.614	0.195	9.879	0.002	1.847 (1.260–2.708)
Oil transportation workers	0.986	0.217	20.754	<0.001	2.682 (1.754–4.100)
Oil refining workers	–0.447	0.251	3.166	0.075	0.640 (0.391–1.046)
Smoking history	-	-	14.044	0.001	-
Previous	0.675	0.200	11.346	0.001	1.964 (1.326–2.908)
Often	0.599	0.191	9.846	0.002	1.820 (1.252–2.646)
Drinking history	-	-	19.421	<0.001	-
Occasional	–0.569	0.185	9.442	0.002	0.566 (0.394–0.814)
Often	0.112	0.213	0.274	0.601	1.118 (0.736–1.699)
Physical exercise	-	-	0.350	0.840	-
Occasional	−0.054	0.160	0.115	0.735	0.947 (0.692–1.297)
Often	0.052	0.221	0.055	0.814	1.053 (0.684–1.623)
BMI	-	-	38.880	<0.001	-
Overweight	0.748	0.176	18.031	<0.001	2.112 (1.496–2.983)
Obesity	1.208	0.194	38.685	<0.001	3.347 (2.287–4.898)

Note: β, regression coefficient; SE, standard error; HR, hazard ratio.

**Table 8:** The risk for hypertension in different occupational stress groups analyzed by a Cox proportional hazards model.

Categorical Variables	Model 1 HR (95% CI)	*p* Value	Model 2 HR (95% CI)	*p* Value
Occupational stress				
Low	1.000	-	1.000	-
Intermediate	1.086 (0.767–1.538)	0.185	0.980 (0.691–1.390)	0.912
High	1.675 (1.161–2.418)	0.006	1.549 (1.072–2.236)	0.020

Note: Model 1, univariate model; Model 2, multivariate model in which type of work, cigarette smoking, alcohol consumption, and BMI were controlled.

**Table 9:** The risk for hypertension of female subjects in different occupational stress groups analyzed by a Cox proportional hazards model.

Categorical Variables	Model 1 HR (95% CI)	*p* Value	Model 2 HR (95% CI)	*p* Value
Occupational stress				
Low	1.000	-	1.000	-
Intermediate	1.075 (0.588–1.965)	0.815	1.060 (0.578–1.945)	0.851
High	2.208 (1.133–4.303)	0.020	2.337 (1.191–4.585)	0.014

Note: Model 1, univariate model; Model 2, multivariate model in which type of work, cigarette smoking, alcohol consumption, and BMI were controlled.

**Table 10:** The risk for hypertension of male subjects in different occupational stress groups analyzed by a Cox proportional hazards model.

Categorical Variables	Model 1 HR (95% CI)	*p* Value	Model 2 HR (95% CI)	*p* Value
Occupational stress				
Low	1.000	-	1.000	-
Intermediate	0.990 (0.645–1.518)	0.962	0.862 (0.558–1.331)	0.502
High	1.263 (0.809–1.970)	0.304	1.269 (0.812–1.984)	0.295

Note: Model 1, univariate model; Model 2, multivariate model in which type of work, cigarette smoking, alcohol consumption, and BMI were controlled.
